# Pubertal Assessment and Growth in Patients With Hemoglobinopathies: A Longitudinal Multicenter Study on the Association With Ferritin Levels

**DOI:** 10.1111/ejh.70075

**Published:** 2025-12-07

**Authors:** J. Dülberg, C. Sanchez, M.‐A. Burckhardt, L. Alacán Friedrich, V. Salow, A. Radauer‐Plank, A. Borgmann‐Staudt, H. Cario, M. Diepold, B. Drexler, L. Infanti, S. Kroiss, N. Dietliker, R. Merki, L. Njue, L. Oevermann, A. Rovó, K. Scheinemann, M. Schneider, M. Balcerek, T. Diesch‐Furlanetto

**Affiliations:** ^1^ Faculty of Medicine University of Basel Basel Switzerland; ^2^ Division of Pediatric Hematology and Oncology University Children's Hospital Basel Basel Switzerland; ^3^ Pediatric Research Centre, University Children's Hospital Basel University of Basel Basel Switzerland; ^4^ Pediatric Endocrinology and Diabetology University Children's Hospital Basel Basel Switzerland; ^5^ Department of Pediatric Oncology and Hematology, Charité‐Universitätsmedizin Berlin Corporate Member of Freie Universität Berlin and Humboldt‐Universität Zu Berlin Berlin Germany; ^6^ Department of Pediatrics and Adolescent Medicine University Medical Centre Ulm Ulm Germany; ^7^ Division of Pediatric Hematology and Oncology, Children's Hospital Inselspital Bern Bern Switzerland; ^8^ Division of Hematology University Hospital Basel Basel Switzerland; ^9^ Department of Pediatric Hematology and Oncology University Children's Hospital Zurich Zurich Switzerland; ^10^ Department of Medical Oncology and Hematology University Hospital Zurich Zurich Switzerland; ^11^ Division of Hematology, Kantonsspital Luzern Lucerne Switzerland; ^12^ Division of Hematology, Inselspital Bern Bern Switzerland; ^13^ Division of Pediatric Hematology and Oncology, Ostschweizer Kinderspital St. Gallen Switzerland; ^14^ Faculty of Health Sciences and Medicine University of Lucerne Lucerne Switzerland; ^15^ Department of Pediatrics, St. Anna Children's Hospital Medical University Vienna Vienna Austria; ^16^ Department of Oncology and University Cancer Center Leipzig University of Leipzig Medical Center Leipzig Germany

**Keywords:** chronic transfusion, hemoglobinopathies, iron overload, puberty

## Abstract

**Objectives:**

Although Advancements in the Treatment of Hemoglobinopathies have Considerably Increased Life Expectancy, Hormonal and Pubertal Development Have Been Continuously Affected by Complications From Transfusion‐Related Iron Overload and Cytotoxic Therapies. This Study Investigated the Association Between Serum Ferritin Levels and Pubertal Progression in Patients With Thalassemia and Sickle Cell Disease (SCD).

**Methods:**

Data Collected From 10 Hospitals in Austria, Germany, and Switzerland From 2012 to 2020 Were Retrospectively Analyzed. We Enrolled 140 Individuals (Median Age: 16.5 Years) With Thalassemia or SCD.

**Results:**

Overall, Delayed Puberty Was Observed in 14.7% (6.7% Females; 21.1% Males) and 13.2% of Patients With Thalassemia and SCD (6.9% Females; 20.8% Males), respectively. Gonadal Insufficiency Was Found in 13.3% and 8.6% of Females With Thalassemia and SCD, Respectively. Abnormal Growth Trajectories Were Observed in 32.5% (28.5% Females; 36.8% Males) and 18.7% of Patients With Thalassemia and SCD (13.3% Females; 23.5% Males), respectively. A Statistically Significant Association Was Found Between Elevated Ferritin Levels and Growth Delays in Patients With Thalassemia. Notably, Tanner Staging Data Were Missing in 80.7% of the Medical Records.

**Conclusions:**

Our Results Indicated the Need for Comprehensive Pubertal Screening and Underscored the Importance of Robust Endocrine Follow‐Up Care in Individuals With Hemoglobinopathies.

**Abbreviations**
AWMF: Working Group of the Scientific Medical Societies
BMI: Body mass index
CCA: Chronic congenital anemia
DACH: Germany, Austria, Switzerland
EC: Erythrocyte concentrates
FSH: Follicle‐stimulating hormone
GnRH: Gonadotropin‐releasing hormone
HRT: Hormone replacement therapy
HSCT: Hematopoietic stem cell transplantation
HU: Hydroxycarbamide
LH: Luteinizing hormone
RKI: Robert Koch Institute
SCD: Sickle cell disease
TM: Thalassemia major

## 1 Introduction

In Europe, thalassemia major (TM) and sickle cell disease (SCD) are classified as rare diseases; however, their prevalence has been increasing owing to migratory patterns. Therapeutic advances—including optimized transfusion protocols, iron chelation strategies, serial monitoring of iron burden, and hydroxyurea (HU) use—have remarkably enhanced survival in affected individuals [1, 2, 3]. Nonetheless, the chronic nature of these disorders requires specialized multidisciplinary care to alleviate disease‐related complications and foster normal growth and developmental outcomes. Puberty is a pivotal stage in physical and psychosocial maturation [4]. Patients with hemoglobinopathies face an elevated risk of delayed or incomplete pubertal development and impaired growth trajectories [5, 6], which might be attributed to several factors, such as chronic disease burden, suboptimal treatment adherence, nutritional deficits, etc. [7, 8, 9]. Transfusion‐related iron overload, particularly in inadequately chelated patients, may lead to iron deposition within the pituitary gland, potentially resulting in hypogonadotropic hypogonadism and other endocrine disorders. This pathophysiological mechanism is particularly pronounced in TM [10, 11]. Notably, the iron distribution pattern differs between SCD and TM: although hepatic iron accumulation is common to both, extrahepatic deposition (e.g., deposition at myocardial and pituitary sites) occurs more frequently in TM [12]. Early recognition and intervention are crucial to ensure normal pubertal development, achieve genetic target height, and prevent long‐term consequences, such as infertility and sexual dysfunction [13, 14]. In addition, early therapeutic measures can markedly alleviate the manifestations of pubertal disorders, such as primary amenorrhea, delayed puberty, and hypogonadotropic hypogonadism; evidence‐based treatment options are available for all these manifestations [15, 16]. Despite the clinical importance of these developmental outcomes, data from European cohorts on pubertal trajectories in patients with hemoglobinopathies remain scarce [17, 18, 19]. The present study investigated pubertal development in patients with TM and SCD across tertiary care centers in Germany, Austria, and Switzerland (DACH region), particularly focusing on their association with serum ferritin levels and iron overload.

## 2 Objectives

### 2.1 Primary Objective

To determine the prevalence of delayed pubertal development in patients with TM and SCD, stratified by the presence or absence of iron overload.

### 2.2 Secondary Objective

To quantify the frequency and completeness of documented pubertal staging, emphasizing the use of standardized assessment criteria, such as Tanner staging.

## 3 Methods

### 3.1 Patient Recruitment

This multicenter retrospective‐prospective cohort study enrolled patients diagnosed with TM or SCD who required red blood cell transfusion across 10 pediatric and adult hematology departments in the DACH region. Patient data were retrospectively collected from January 2012 to May 2019 and prospectively documented from June 2019 to December 2020. Additional structured onsite surveys were conducted during routine clinical visits, along with follow‐up data regarding Tanner stages, weight, height and hormonal data. For patients who joined the cohort later, data were retrospectively collected from 2012 up to their inclusion date. The inclusion criteria encompassed individuals aged 12–25 years during the study period. For patients who turned 12 years old during this period, earlier medical records were retrospectively included to provide additional data related to their transition from childhood to adolescence. Data acquisition concluded at the end of 2020, upon the patient reaching 25 years of age or at loss to follow‐up.

Participation required sufficient German language proficiency and the ability to provide written informed consent. For underaged participants, consent was obtained from their legal guardians. All data were pseudonymized. Ethical approval for the study protocol was acquired from Charité‐Universitätsmedizin Berlin (EA2/017/18), the Medical University Vienna (EK Nr:2264/2018), and the Ethics Committee of Northwestern/Central Switzerland (EKNZ 2018–02044).

### 3.2 Variables and Outcomes

The patients were stratified by primary diagnosis (TM or SCD). To isolate the effect of cumulative transfusion‐related iron overload on pubertal development, only the data up to the start of HU therapy or the year of hematopoietic stem cell transplantation (HSCT) were included to reduce confounding by disease‐modifying interventions. The following clinical variables were extracted from the patients' medical records: demographics (age, sex, migration background, and diagnostic category), treatment history (transfusion volumes and chelation status), clinical parameters (anthropometric measurements, pubertal staging, and annual laboratory values), and survey measures (sociodemographics, self‐reported age at menarche/ejaculation, hormone therapy use, and menopausal symptoms).

Annual transfusion burden was quantified by the cumulative number of erythrocyte concentrate (EC) units received. According to national guidelines of the Association of the Scientific Medical Societies in Germany (AWMF), iron overload was defined as either transfusion exposure ≥ 10 EC or serum ferritin concentration > 1000 μg/L [20, 21]. The follow‐up period was defined as beginning after the first year of study inclusion and continuing until the last available year of documented data.

### 3.3 Pubertal Development Assessment

**FIGURE 1:**
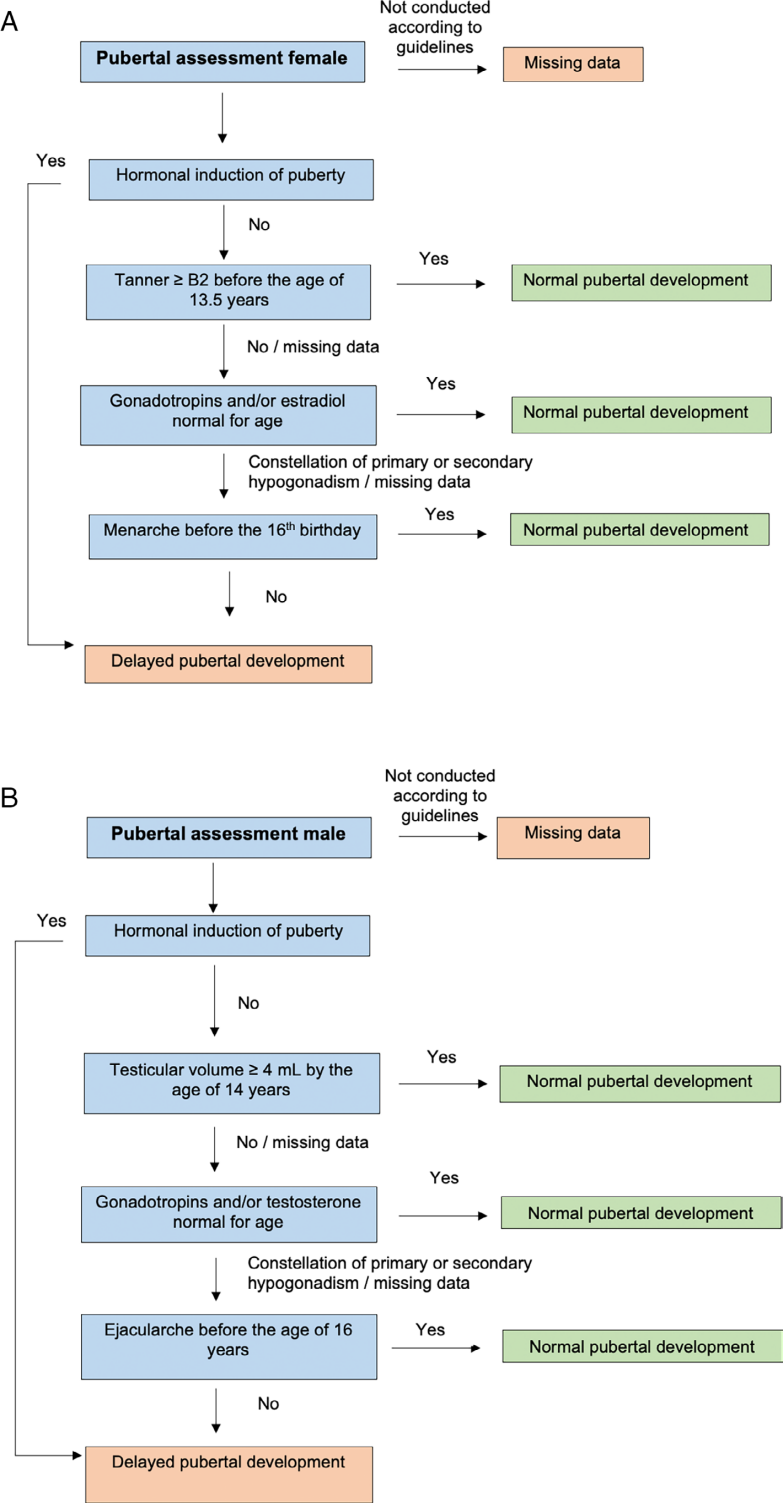
(A) Algorithm used to evaluate female puberty. (B) Algorithm used to evaluate male puberty.

Hormone treatments—including pubertal induction, hormone replacement therapy (HRT), and oral contraceptive use—were recorded. Normal pubertal development was defined as menarche between the ages of 11–16 and/or the attainment of Tanner stage ≥ B2 by the age of 13.5 for females and testicular volume ≥ 4 mL by the age of 14 for males. These criteria aligned with the AWMF guidelines used in the DACH region on delayed puberty and hypogonadism [22, 23, 24]. Cases that did not meet these benchmarks or required pharmacological induction were classified as experiencing delayed pubertal development. Oral contraceptive use was considered indicative of early menarche. Primary amenorrhea was defined as the absence of menarche by the completion of the 15th year of life [25]. In HRT cases with insufficient clinical detail, hypogonadotropic hypogonadism was presumed. Figure 1A,B illustrate the pubertal staging protocols.

Height, weight, and body mass index (BMI) were evaluated against age‐standardized percentile curves published by the Robert Koch Institute (RKI) [26]. These national reference datasets, widely used within DACH‐region hospitals, incorporate a slightly higher proportion of individuals with migrant backgrounds compared to the WHO standards (18.8% vs. 15.3%) [27, 28], thereby offering a more efficient representative baseline for the hemoglobinopathy population. Migration status was assigned to a participant when either the patient or at least one of their parents originated from outside the DACH region. Educational level was determined using the International Standard Classification of Education (ISCED 2011), categorized as low (ISCED 1–2) or medium/high (ISCED 3–8) based on questionnaire responses [29].

### 3.4 Statistical Methods

The patient's baseline characteristics were summarized as frequency (%) for categorical variables and median (interquartile range [IQR]) for continuous variables. Analyses were based solely on complete cases. A logistic regression was conducted, with delayed pubertal development as the outcome and nine potential risk factors included in our initial analysis: ferritin levels (mean value over observation period), age, sex, disease type, height z‐score, BMI z‐score, total amount of EC administered during the study period, hemoglobin levels, and region of origin. Linear regression was performed with log‐transformed serum ferritin as the outcome and height/weight/BMI z‐scores, sex, and disease as predictors to assess the association between serum ferritin and the other parameters. Forward–backward variable selection was employed to determine the most parsimonious model. Variables with *p* < 0.25 in univariate regression were selected for the stepwise elimination process. Analyses were conducted using the statistical software R (version 4.2.1, R Development Core Team, Vienna, Austria) [30].

## 4 Results

### 4.1 Patient Characteristics

A total of 140 patients were enrolled in the study, including 34 (24.3%) and 106 (75.7%) patients with TM and SCD, respectively. Follow‐up data were available for 91 patients (65%), with a median follow‐up duration of 4.0 (IQR: 3.0–5.3) years. Follow‐up was discontinued for 45 and four patients with TM and SCD, respectively, due to the initiation of HU therapy or HSCT.

The median age at the time of inclusion was 17.0 (IQR: 14.0–18.0) and 16.0 (IQR: 14.0–20.0) years for females and males with TM and 17.0 (IQR: 14.0–20.0) and 16.0 (IQR: 14.0–18.5) years for females and males with SCD, respectively. All patients had migrated from outside the DACH region, predominantly from Sub‐Saharan Africa (42.6%) and Western Asia (23.5%). Data collection was distributed across Germany (64.3%), Austria (18.6%), and Switzerland (17.1%).

At follow‐up conclusion, 31.4% (44/140) of the patients had received ≥ 10 EC units, and 95.4% (42/44) of them were on chelation therapy. Patients with TM and SCD received a median of 68.5 (IQR: 44.0–115.0) and 5.0 (IQR: 1.5–12.0) EC units, respectively. Iron overload, defined as serum ferritin levels > 1000 μg/L, was observed in 17.8% (25/140) of patients, with a markedly higher prevalence in those with TM (67.6%, 23/34) than in those with SCD (1.9%, 2/106). Among the patients with iron overload, 52% (13/25) underwent liver iron quantification via T2*‐weighted magnetic resonance imaging. The patients' baseline characteristics are presented in Table 1.

**TABLE 1:** Patient characteristics.

Gender	SCD			Thalassemia		
	Overall *N* = 106^a^	Female *N* = 58^a^	Male *N* = 48^a^	Overall *N* = 34^a^	Female *N* = 15^a^	Male *N* = 19^a^
Age at inclusion (years)						
Median [Q1, Q3]	17.0 [14.0, 20.0]	17.0 [14.0, 20.0]	16.0 [14.0, 18.5]	16.0 [14.0, 19.0]	17.0 [14.0, 18.0]	16.0 [14.0, 20.0]
Min, Max	(12.0, 25.0)	(12.0, 25.0)	(13.0, 25.0)	(12.0, 25.0)	(12.0, 24.0)	(13.0, 25.0)
Follow‐up (years)						
Median [Q1, Q3]	1.0 [1.0, 3.0]	1.0 [1.0, 3.0]	1.0 [1.0, 3.0]	10.0 [7.0, 10.0]	10.0 [3.5, 10.0]	10.0 [8.0, 10.0]
Min, Max	(1.0, 10.0)	(1.0, 9.0)	(1.0, 10.0)	(1.0, 10.0)	(3.0, 10.0)	(1.0, 10.0)
Missing due to HSCT/HU	45	26	19	4	3	1
Migration background						
Yes	98 (92.5%)	53 (91.4%)	45 (93.8%)	31 (91.2%)	13 (86.7%)	18 (94.7%)
Missing	8 (7.5%)	5 (8.6%)	3 (6.3%)	3 (8.8%)	2 (13.3%)	1 (5.3%)
Educational level						
High (ISCED 6–8)	28 (26.4%)	20 (34.5%)	8 (16.7%)	5 (14.7%)	3 (20.0%)	2 (10.5%)
Medium (ISCED 3–5)	13 (12.3%)	7 (12.1%)	6 (12.5%)	7 (20.6%)	2 (13.3%)	5 (26.3%)
Low (ISCED 1–2)	12 (11.3%)	7 (12.1%)	5 (10.4%)	4 (11.8%)	1 (6.7%)	3 (15.8%)
Missing	53 (50.0%)	24 (41.4%)	29 (60.4%)	18 (52.9%)	9 (60.0%)	9 (47.4%)
EC units						
Median [Q1, Q3]	5.0 [1.5, 12.0]	4.0 [1.0, 12.0]	6.0 [2.0, 8.0]	68.5 [44.0, 115.0]	70.0 [44.0, 115.0]	67.0 [36.0, 122.0]
Min, Max	(1.0, 527.0)	(1.0, 527.0)	(1.0, 139.0)	(11.0, 651.0)	(11.0, 331.0)	(18.0, 651.0)
Missing	70	39	31			
Transfusion therapy (> 10 EC)						
Yes	10 (9.4%)	6 (10.3%)	4 (8.3%)	34 (100.0%)	15 (100.0%)	19 (100.0%)
No	96 (90.6%)	52 (89.7%)	44 (91.7%)	0 (0.0%)	0 (0.0%)	0 (0.0%)
Iron overload						
Yes	2 (1.9%)	1 (1.7%)	1 (2.1%)	23 (67.6%)	11 (73.3%)	12 (63.2%)
No	104 (98.1%)	57 (98.3%)	47 (97.9%)	11 (32.4%)	4 (26.7%)	7 (36.8%)
Iron chelation						
Yes	9 (8.5%)	5 (8.6%)	4 (8.3%)	33 (97.1%)	14 (93.3%)	19 (100.0%)
No	96 (90.6%)	53 (91.4%)	43 (89.6%)	1 (2.9%)	1 (6.7%)	0 (0.0%)
Missing	1 (0.9%)	0 (0.0%)	1 (2.1%)	0 (0.0%)	0 (0.0%)	0 (0.0%)
HU^3^						
Yes	103 (97.2%)	57 (98.3%)	46 (95.8%)	8 (23.5%)	3 (20.0%)	5 (26.3%)
No	3 (2.8%)	1 (1.7%)	2 (4.2%)	26 (76.5%)	12 (80.0%)	14 (73.7%)
Age at the start of HU administration (years)						
Median [Q1, Q3]	8.7 [6.1, 11.6]	8.9 [6.5, 11.8]	8.3 [6.0, 11.1]	12.6 [12.3, 17.8]	15.1 [11.7, 17.8]	12.5 [12.4, 16.5]
Min, Max	(0.8, 17.8)	(0.8, 17.3)	(1.2, 17.8)	(11.7, 20.4)	(11.7, 17.8)	(12.3, 20.4)
Missing	9	3	6	27	12	15
HSCT^2^						
Yes	26 (24.5%)	9 (15.5%)	17 (35.4%)	9 (26.5%)	5 (33.3%)	4 (21.1%)
No	79 (74.5%)	49 (84.5%)	30 (62.5%)	25 (73.5%)	10 (66.7%)	15 (78.9%)
Missing	1 (0.9%)	0 (0.0%)	1 (2.1%)	0 (0.0%)	0 (0.0%)	0 (0.0%)
Age at HSCT initiation (years)						
Median [Q1, Q3]	14.0 [11.0, 17.0]	13.0 [11.0, 18.0]	14.0 [12.0, 16.0]	8.5 [3.5, 11.5]	9.0 [2.0, 11.0]	8.0 [5.0, 12.0]
Min, Max	(7.0, 22.0)	(7.0, 20.0)	(9.0, 22.0)	(1.0, 15.0)	(1.0, 15.0)	(5.0, 12.0)
Missing	80	49	31	26	10	16
Recruitment country						
Austria	19 (17.9%)	11 (19.0%)	8 (16.7%)	7 (20.6%)	3 (20.0%)	4 (21.1%)
Germany	69 (65.1%)	36 (62.1%)	33 (68.8%)	21 (61.8%)	9 (60.0%)	12 (63.2%)
Switzerland	18 (17.0%)	11 (19.0%)	7 (14.6%)	6 (17.6%)	3 (20.0%)	3 (15.8%)

Abbreviations: EC, Erythrocyte concentrate; HSCT, Hematopoietic stem cell transplantation; HU, Hydroxyurea; ISCED, International Standard Classification of Education; Q1, First quadrant; Q3, Third quadrant.

^a^ *n* (%), as applicable.

### 4.2 Pubertal Development and Induction

Normal pubertal onset was observed in 76.5% (26/34) and 78.3% (83/106) of patients with TM and SCD, respectively. Delayed puberty was identified in 14.7% (5/34, stratified as 6.7% in females and 21.1% in males) and13.2% (14/106, stratified as 6.9% in females and 20.8% in males) of patients with TM and SCD, respectively, based on a standardized pubertal assessment algorithm (Figure 1A,B).

Menarche or ejacularche was documented in 62.1% (87/140) of patients, and 94.2% (82/87) of them had already experienced the event. Among females, the median age at menarche was 13.8 (IQR: 12.9–14.0) years and 13.1 (IQR: 12.3–14.3) years for females with TM and SCD, respectively. Delayed menarche was documented in one female with TM and none with SCD. Ejacularche was recorded in 1 of 19 male patients with TM at the age of 18.0 years and in 4 of 48 male patients with SCD at a median age of 12.5 (IQR: 11.0–13.5) years.

Tanner staging data were available for only 19.3% (27/140) of patients, predominantly from pediatric hospitals (85.2%, 23/27) rather than adult centers (14.8%, 4/27). Based on Tanner staging, hormone profiles, and HRT documentation, gonadal insufficiency was reported in 13.3% (2/15) and 8.6% (5/58) of females with TM and SCD, respectively, but in none of the male patients (Table 2). Pubertal induction therapy was administered to 17.6% (6/34) of patients with TM (20% females and 15.8% males) and 3.8% (4/106) of patients with SCD (3.4% females and 4.2% males). HRT was required in 3.6% (5/140) of the patients, with disproportionate use in TM (11.8%) versus SCD (0.9%). Three out of the five patients who received HRT were overloaded with iron. Hormonal contraception was recorded in 4.1% (3/73) of female patients (Table 3).

**TABLE 2:** Pubertal development.

Gender	SCD			Thalassemia		
	Overall *N* = 106^a^	Female *N* = 58^a^	Male *N* = 48^a^	Overall *N* = 34^a^	Female *N* = 15^a^	Male *N* = 19^a^
Regular pubertal onset						
Yes	83 (78.3%)	53 (91.4%)	30 (62.5%)	26 (76.5%)	15 (100.0%)	11 (57.9%)
No	4 (3.8%)	0 (0.0%)	4 (8.3%)	2 (5.9%)	0 (0.0%)	2 (10.5%)
Missing	19 (17.9%)	5 (8.6%)	14 (29.2%)	6 (17.6%)	0 (0.0%)	6 (31.6%)
Delayed pubertal development^b^						
Yes	14 (13.2%)	4 (6.9%)	10 (20.8%)	5 (14.7%)	1 (6.7%)	4 (21.1%)
No	65 (61.3%)	46 (79.3%)	19 (39.6%)	23 (67.6%)	14 (93.3%)	9 (47.4%)
Missing	27 (25.5%)	8 (13.8%)	19 (39.6%)	6 (17.6%)	0 (0.0%)	6 (31.6%)
Spontaneous/induced						
Spontaneous	82 (77.4%)	51 (87.9%)	31 (64.6%)	21 (61.8%)	11 (73.3%)	10 (52.6%)
Induced	4 (3.8%)	2 (3.4%)	2 (4.2%)	6 (17.6%)	3 (20.0%)	3 (15.8%)
Missing	20 (18.9%)	5 (8.6%)	15 (31.3%)	7 (20.6%)	1 (6.7%)	6 (31.6%)
Menarche						
Yes	49 (46.2%)	49 (84.5%)	NA [NA, NA]	14 (41.2%)	14 (93.3%)	NA [NA, NA]
No	2 (1.9%)	2 (3.4%)	(Inf, −Inf)	1 (2.9%)	1 (6.7%)	(Inf, ‐Inf)
Missing	55 (51.9%)	7 (12.1%)	48	19 (55.9%)	0 (0.0%)	19
Menarche age						
Median [Q1, Q3]	13.1 [12.3, 14.3]	13.1 [12.3, 14.3]	NA [NA, NA]	13.8 [12.9, 14.0]	13.8 [12.9, 14.0]	NA [NA, NA]
Min, Max	(10.0, 16.0)	(10.0, 16.0)	(Inf, ‐Inf)	(10.9, 16.2)	(10.9, 16.2)	(Inf, ‐Inf)
Missing	63	15	48	23	4	19
Menarche type						
Premature	0 (0.0%)	0 (0.0%)	NA [NA, NA]	0 (0.0%)	0 (0.0%)	NA [NA, NA]
Normal	43 (40.6%)	43 (74.1%)	NA [NA, NA]	10 (29.4%)	10 (66.7%)	NA [NA, NA]
Delayed	0 (0.0%)	0 (0.0%)	NA [NA, NA]	1 (2.9%)	1 (6.7%)	NA [NA, NA]
Missing	63 (59.4%)	15 (25.9%)	48	23 (67.6%)	4 (26.7%)	19
Ejacularche						
Yes	16 (15.1%)	NA [NA, NA]	16 (33.3%)	3 (8.8%)	NA [NA, NA]	3 (15.8%)
No	1 (0.9%)	NA [NA, NA]	1 (2.1%)	1 (2.9%)	NA [NA, NA]	1 (5.3%)
Missing	89 (84.0%)	58	31 (64.6%)	30 (88.2%)	15	15 (78.9%)
Ejacularche age						
Median [Q1, Q3]	12.5 [11.0, 13.5]	NA [NA, NA]	12.5 [11.0, 13.5]	18.0 [18.0, 18.0]	NA [NA, NA]	18.0 [18.0, 18.0]
Min, Max	(10.0, 14.0)	(Inf, ‐Inf)	(10.0, 14.0)	(18.0, 18.0)	(Inf, ‐Inf)	(18.0, 18.0)
Missing	102	58	44	33	15	18
Ejacularche type						
Premature	2 (1.9%)	NA [NA, NA]	2 (4.2%)	0 (0.0%)	NA [NA, NA]	0 (0.0%)
Normal	2 (1.9%)	NA [NA, NA]	2 (4.2%)	0 (0.0%)	NA [NA, NA]	0 (0.0%)
Delayed	0 (0.0%)	NA [NA, NA]	0 (0.0%)	1 (2.9%)	NA [NA, NA]	1 (5.3%)
Missing	102 (96.2%)	58	44 (91.7%)	33 (97.1%)	15	18 (94.7%)
Gonadal insufficiency						
Yes	5 (4.7%)	5 (8.6%)	0 (0.0%)	2 (5.9%)	2 (13.3%)	0 (0.0%)
No	65 (61.3%)	37 (63.8%)	28 (58.3%)	22 (64.7%)	11 (73.3%)	11 (57.9%)
Missing	36 (34.0%)	16 (27.6%)	20 (41.7%)	10 (29.4%)	2 (13.3%)	8 (42.1%)

Abbreviations: Q1, First quadrant; Q3, Third quadrant; SCD, Sickle cell disease.

^a^ *n* (%), as applicable.

^b^ As defined in Figure 1.

**TABLE 3:** Hormone therapy application.

Gender	SCD			Thalassemia		
	Overall *N* = 106^a^	Female *N* = 58^a^	Male *N* = 48^a^	Overall *N* = 34^a^	Female *N* = 15^a^	Male *N* = 19^a^
Hormone therapy						
Yes	8 (7.5%)	6 (10.3%)	2 (4.2%)	7 (20.6%)	4 (26.7%)	3 (15.8%)
No	93 (87.7%)	50 (86.2%)	43 (89.6%)	27 (79.4%)	11 (73.3%)	16 (84.2%)
Missing	5 (4.7%)	2 (3.4%)	3 (6.3%)	0 (0.0%)	0 (0.0%)	0 (0.0%)
Hormone replacement therapy						
Yes	1 (0.9%)	1 (1.7%)	0 (0.0%)	4 (11.8%)	2 (13.3%)	2 (10.5%)
No	99 (93.4%)	54 (93.1%)	45 (93.8%)	30 (88.2%)	13 (86.7%)	17 (89.5%)
Missing	6 (5.7%)	3 (5.2%)	3 (6.3%)	0 (0.0%)	0 (0.0%)	0 (0.0%)
Contraception						
Yes	2 (1.9%)	2 (3.4%)	0 (0.0%)	1 (2.9%)	1 (6.7%)	0 (0.0%)
No	98 (92.5%)	53 (91.4%)	45 (93.8%)	33 (97.1%)	14 (93.3%)	19 (100.0%)
Missing	6 (5.7%)	3 (5.2%)	3 (6.3%)	0 (0.0%)	0 (0.0%)	0 (0.0%)

Abbreviation: SCD, Sickle cell disease.

^a^ *n* (%).

### 4.3 Height, Weight, and BMI

**FIGURE 2:**
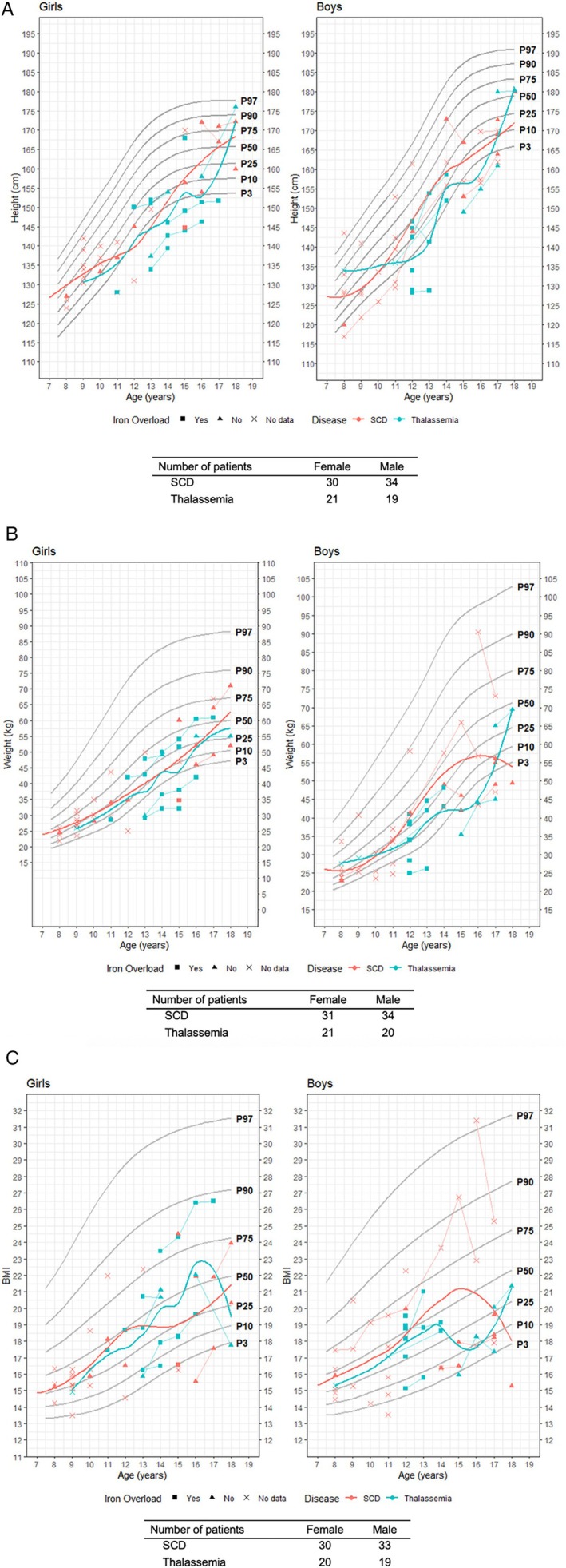
Growth data for patients with thalassemia major and SCD, stratified by sex. (A) Height versus age, (B) weight versus age, and (C) BMI versus age. The number of patients with available data is shown. Color distinguishing sex. Symbols were linked when the follow‐up data of a patient was available. Symbols showed if iron overload was present in the patients, with “x” indicating that no data regarding iron overload was available for a patient. SCD: Sickle cell disease.

In patients with TM, 26.5% (9/34) had heights below the age‐specific 3rd percentile for height (26.7% females, 26.3% males). Furthermore, 14.7% (5/34; 13.3% females, 15.8% males) of the patients had low weight (< 3rd percentile), and 5.9% (2/34; 6.7% females, 5.3% males) of the patients exhibited reduced BMI. Contrarily, among patients with SCD, 7.5% (8/106), 23% (9/106), and 6.6% (7/106) fell below the 3rd percentile for height (5.2% females, 10.4% males), weight (3.4% females, 14.6% males), and BMI (8.6% females, 4.2% males), respectively (Figure 2A–C).

A logistic regression model was developed to investigate the predictors of delayed pubertal development (Table S1), incorporating the following variables: mean ferritin levels (2012–2020), age, sex, disease type, height z‐score, BMI z‐score, cumulative number of EC units received, hemoglobin levels, and region of origin. The application of a forward–backward selection algorithm yielded a final model that retained sex, ferritin concentration, and height z‐score as independent variables. The weight z‐score was excluded owing to multicollinearity with BMI and height z‐score. None of the included predictors showed a statistically significant association with delayed puberty.

**FIGURE 3:**
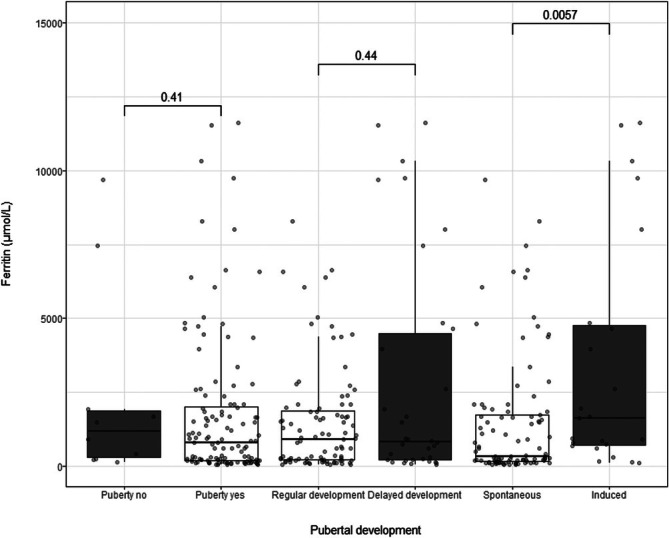
Pubertal development versus ferritin levels in patients born after 2000 (age range: 12–21 years during the observation period). Pubertal development is split into whether regular pubertal onset is yes/no, regular/delayed pubertal development, and spontaneous/induced puberty. *p*‐value for the correlation between ferritin and pubertal development is shown.

In a secondary analysis, linear regression was performed using log‐transformed ferritin levels as the dependent variable and height/weight/BMI z‐scores, sex, and disease type as the independent variables. Statistically significant associations were observed for disease type (TM vs. SCD; β = 2.0, 95% confidence interval (CI): 1.4–2.7, *p* < 0.0001) and height z‐score (β = −0.22, 95% CI: −0.42 to −0.01, *p* = 0.037; Table S2). Figure 3 illustrates the correlation between pubertal development and ferritin levels.

## 5 Discussion

This study evaluated pubertal development in patients with TM and SCD treated at 10 tertiary centers across the DACH region. Delayed pubertal development was observed in 14.7% of patients with TM (6.7% females and 21.1% males) and 13.2% of patients with SCD (6.9% females and 20.8% males), based on various pubertal milestones. HRT was administered in 3.6% of the overall cohort (11.8% TM and 0.9% SCD), whereas puberty induction was required in 7.1% (17.6% TM and 3.8% SCD).

Previous studies reported high rates of delayed or failed puberty in patients with TM and SCD, affecting up to 80% of patients with TM [31, 32, 33]. More recent data from France indicated delayed puberty in 16% of patients with TM [19], consistent with our findings and reflecting improved disease management. In our cohort, 95.4% of patients with iron overload received chelation therapy, likely contributing to better pubertal outcomes, particularly in those with TM.

The median age at menarche in females with TM and SCD was similar to healthy controls (12–13.5 years), contrasting with previously reported delays [32, 34, 35]. Compared with German and the US reference populations [36, 37], however, menarche occurred later, possibly reflecting the cohort's migratory background. Semen analysis prior to HU initiation is advised. Nonetheless, in our cohort, HU therapy was commenced at the median ages of 12.6 and 8.7 years in patients with TM and SCD, respectively. Hence, semen data could not be collected, as most patients were prepubertal at the initiation of therapy.

Literature search yielded no data on ejacularche in patients with TM or SCD. Herein, one male patient with TM reported ejacularche at the age of 18.0 years, considerably later compared to the patients with SCD (median age: 12.5 years). This discrepancy might be associated with more frequent transfusions and higher iron overload in patients with TM than in those with SCD (100% vs. 9.4% and 67.6% vs. 1.9%, respectively). However, owing to the limited sample size, definitive conclusions could not be drawn, highlighting the need for further research. Both menarche and ejacularche—referring to the first conscious ejaculation, as opposed to spermaturia, which was not evaluated in this study—are milestones that are typically well remembered, even retrospectively [38, 39]. Therefore, data collected using questionnaires or medical history entries are considered reliable. The high rate of missing data indicates that ejaculatory history remains underreported in clinical practice.

Delayed puberty was more frequent in males than in females in TM and SCD (TM: 21.1% in males vs. 6.7% in females and SCD: 20.8% in males vs. 6.9% in females). Unlike previous studies linking ferritin to hypogonadism and growth impairment [40, 41], our analysis revealed no significant association. This may reflect limited follow‐up owing to high patient mobility and incomplete longitudinal data. Overall, delayed puberty appears multifactorial rather than solely related to iron overload.

The observed sex‐based disparity might reflect varying clinical manifestations of hypogonadotropic hypogonadism, which tend to be more pronounced in males—such as absent voice deepening, small stature, or lack of facial hair—and less apparent in females. Owing to the small sample size in our study, larger‐scale investigations are warranted to validate our findings and further elucidate underlying mechanisms.

The lack of documented Tanner stages was noted. In pediatric care settings, patients are often cared for by small, continuous teams until the patients transition to adult care. We hypothesized that Tanner staging is acknowledged by healthcare providers but not formally recorded. Nevertheless, precise documentation remains essential for objective clinical assessment. Moreover, documentation of Tanner stages is crucial for the evaluation of pubertal development and identification of delays, thereby informing appropriate therapeutic interventions.

In addition to Tanner staging and the onset of menarche or ejaculation, physical growth indicators provide valuable insights into pubertal progression. Based on age‐specific percentile curves for height, weight, and BMI proposed by RKI, adolescents with TM or SCD predominantly fell within the 3rd and 50th percentiles, reflecting growth delay. Although these curves are derived from data on the general German population—which includes a relatively higher proportion of individuals with migrant backgrounds than the WHO references—they do not fully capture ethnic‐specific variations. As most patients in the cohort came from sub‐Saharan Africa and West Asia, a lower target height was possible [42].

Patients with TM tended to have lower height and weight than those with SCD, corroborating established literature [7, 43]. Both groups exhibited delayed pubertal growth spurt at 18 years of age, which was consistent with the findings from other studies [8, 9]. Consistent with previous studies [44], elevated ferritin levels were the main predictors of reduced height, particularly in males with TM. The observed delay in pubertal growth might also be influenced by the timing of hormone therapy initiation following the recognition of growth impairment.

Continuous monitoring and documentation of iron status, pubertal development, and growth parameters in patients with TM and SCD are crucial to ensure timely and individualized treatment. In our cohort, only 52% (13/25) of patients with iron overload underwent liver iron quantification by T2*‐weighted magnetic resonance imaging, highlighting a gap between AWMF guidelines [21] and clinical practice. Annual Tanner staging and liver iron assessment were inconsistently implemented across the centers.

Despite these documentation gaps, HRT use was lower than expected, and age at menarche in patients with TM and SCD was earlier than previously reported. This finding reflected a high standard of care, though limited Tanner stage documentation and infrequent liver iron assessments indicate room for improvement. As many causes of delayed pubertal development are preventable and treatable, greater adherence to the established guidelines is crucial in daily clinical practice.

Puberty is a challenging period for all adolescents [45]. Addressing the fears and concerns of adolescents and their parents is crucial, particularly when chronic illness affects pubertal development. An open discussion facilitates timely treatment initiation and supports normal developmental progression. Furthermore, interdisciplinary care involving hematologists, endocrinologists, psychologists, and social workers promotes adherence, ensures continuity during transition to adult care, and fosters autonomy in medical decision‐making [46].

Nevertheless, iron overload is just one of several factors that can negatively affect the onset of puberty and fertility [47]. With increasing migration and a rising number of refugees from Africa, Asia, and the Middle East, hemoglobinopathies are becoming more prevalent in Central Europe and the US [48, 49]. Consequently, healthcare providers in high‐income countries need to have greater awareness of these diseases and their aspects.

This study is limited by missing data, primarily due to COVID‐19–related disruptions and high patient mobility during the transition from pediatric to adult care or migration. These challenges led to considerable loss to follow‐up, reducing statistical power and highlighting the need for national and international registries. Lifestyle factors, such as substance use, which might influence pubertal development, were not evaluated. In addition, given that evaluation of pituitary iron deposition is not a standard imaging practice according to the current guidelines, the study did not include this assessment. As a result, data on possible growth hormone deficiency in patients below the third

Percentile is lacking. Finally, the exclusion of patients receiving HU therapy due to the assumption that the treatment influenced pubertal development may have introduced selection bias. Wang et al. [50] found no difference in height, weight, or growth velocity between children before and during HU treatment. They also observed no adverse effect on pubertal development.

Despite these limitations, the study highlights the importance of early recognition and treatment of delayed puberty in patients with hemoglobinopathies. Adherence to screening protocols—particularly Tanner stage documentation—is crucial.

In conclusion, delayed puberty was observed in 13.6% of patients with TM and SCD in the DACH region, predominantly affecting males with TM. Although the standards of care were generally high, the implementation of key assessments remained inconsistent. Routine monitoring, including Tanner staging and liver iron quantification, is crucial for improving pubertal development and fertility outcomes in this population.

## Author Contributions

Conceptualization: Magdalena Balcerek, Anja Borgmann‐Staudt, Marie Anne Burkhardt, Tamara Diesch‐Furlanetto, and Jill Dülberg. Data collection: Lucia Alacán Friedrich, Jill Dülberg, Anne Radauer‐Plank, Vivienne Salow, Nadja Dietliker, Miriam Diepold, Beatrice Drexler, Laura Infanti, Sabine Kroiss, Ramona Merki, Linet Njue, Alicia Rovó, Kathrin Scheinemann, and Monika Schneider. Data analysis: Carlos Sanchez. Manuscript original draft: Jill Dülberg, Tamara Diesch‐Furlanetto, and Magdalena Balcerek. Supervision: Tamara Diesch‐Furlanetto and Magdalena Balcerek. All coauthors reviewed the manuscript.

## Funding

The data collection and statistical analysis from Switzerland were supported by funding from the Bangerter Rhyner Foundation and the Stiftung für Hämatologische Forschung Basel. Magdalena Balcerek was supported by the Clinician Scientist Program funded by the Berlin Institute of Health (BIH) of the Charité‐Universitätsmedizin Berlin. These organizations provided the financial resources necessary to facilitate the research, covering the costs associated with patient data collection, survey administration, and subsequent statistical analysis.

## Ethics Statement

Ethical approval for the study protocol was acquired from Charité‐Universitätsmedizin Berlin (EA2/017/18), the Medical University Vienna (EK Nr:2264/2018), and the Ethics Committee of Northwestern/Central Switzerland (EKNZ 2018–02044).

## Consent

Written informed consent was obtained from all patients and legal guardians for underaged patients.

## Conflicts of Interest

The authors declare no conflicts of interest.

## Supporting information


**Table S1:** Logistic regression: Relation between delayed pubertal development vs. sex, ferritin, and height z‐score.
**Table S2:** Linear regression: ferritin levels vs. disease, BMI z‐score, and height z‐score.

## Data Availability

The data set from the study is held securely in coded form at the Charité, Berlin, Germany. The data underlying this article will be shared on reasonable request to the corresponding authors after granting the prespecified criteria for confidential access.
