# Supplementary material for: Pubertal Assessment and Growth in Patients With Hemoglobinopathies: A Longitudinal Multicenter Study on the Association With Ferritin Levels
Source: Eur J Haematol. 2025 Dec 7;116(3):276–89. doi: 10.1111/ejh.70075 (PMC12861717; doi:10.1111/ejh.70075)
Supplement: Supplementary file 1 — Table S1: Logistic regression: Relation between delayed pubertal development vs. sex, ferritin, and height z‐score. Table S2: Linear regression: ferritin levels vs. disease, BMI z‐score, and height z‐score. [file EJH-116-276-s001.pdf]

SUPPLEMENTARY TABLES

TABLE S1: Logistic regression: Association between delayed pubertal development vs. sex, ferritin, and height z-score

**Characteristic**	**OR**	**95% CI**	***p*-value**
(Intercept)	0.00	0.00–0.06	0.033
Sex			
Female	—	—	
Male	9.32	0.48–1 470	0.21
Ferritin	1.00	1.00–1.00	0.22
Height z-score	0.29	0.01–1.66	0.30

Abbreviations: CI, Confidence interval; OR, Odds ratio

TABLE S2: Linear regression: Ferritin vs. disease, BMI z-score, and height z-score

**Characteristic**	**Beta**	**95% CI**	***P*-value**
(Intercept)	5.0	4.5–5.5	<0.0001
Disease			
SCD	—	—	
Thalassemia	2.0	1.4–2.7	<0.0001
BMI z-score	0.22	−0.04–0.48	0.091
Height z-score	−0.22	−0.42–0.01	0.037

Abbreviation: CI, Confidence interval; SCD, sickle cell disease; BMI, Body mass index
